# Modification of targets related to the Entner–Doudoroff/pentose phosphate pathway route for methyl-d-erythritol 4-phosphate-dependent carotenoid biosynthesis in *Escherichia coli*

**DOI:** 10.1186/s12934-015-0301-x

**Published:** 2015-08-12

**Authors:** Chun Li, Lan-Qing Ying, Sha-Sha Zhang, Nan Chen, Wei-Feng Liu, Yong Tao

**Affiliations:** CAS Key Laboratory of Microbial Physiological and Metabolic Engineering, Institute of Microbiology, Chinese Academy of Sciences, Beijing, People’s Republic of China; School of Life Science, University of Chinese Academy of Sciences, Beijing, People’s Republic of China

**Keywords:** Carotenoid biosynthesis, Methyl-d-erythritol 4-phosphate pathway, Genetic targets, Central metabolic pathways, *Escherichia coli*, Balance of precursors

## Abstract

**Background:**

In engineered strains of *Escherichia coli*, bioconversion efficiency is determined by not only metabolic flux but also the turnover efficiency of relevant pathways. Methyl-d-erythritol 4-phosphate (MEP)-dependent carotenoid biosynthesis in *E. coli* requires efficient turnover of precursors and balanced flux among precursors, cofactors, and cellular energy. However, the imbalanced supply of glyceraldehyde 3-phosphate (G3P) and pyruvate precursors remains the major metabolic bottleneck. To address this problem, we manipulated various genetic targets related to the Entner–Doudoroff (ED)/pentose phosphate (PP) pathways. Systematic target modification was conducted to improve G3P and pyruvate use and rebalance the precursor and redox fluxes.

**Results:**

Carotenoid production was improved to different degrees by modifying various targets in the Embden–Meyerhof–Parnas (EMP) and ED pathways, which directed metabolic flux from the EMP pathway towards the ED pathway. The improvements in yield were much greater when the MEP pathway was enhanced. The coordinated modification of ED and MEP pathway targets using gene expression enhancement and protein coupling strategies in the *pgi* deletion background further improved carotenoid synthesis. The fine-tuning of flux at the branch point between the ED and PP pathways was important for carotenoid biosynthesis. Deletion of *pfk*AB instead of *pgi* reduced the carotenoid yield. This suggested that anaplerotic flux of G3P and pyruvate might be necessary for carotenoid biosynthesis. Improved carotenoid yields were accompanied by increased biomass and decreased acetate overflow. Therefore, efficient use of G3P and pyruvate precursors resulted in a balance among carotenoid biosynthesis, cell growth, and by-product metabolism.

**Conclusions:**

An efficient and balanced MEP-dependent carotenoid bioconversion strategy involving both the ED and PP pathways was implemented by the coordinated modification of diverse central metabolic pathway targets. In this strategy, enhancement of the ED pathway for efficient G3P and pyruvate turnover was crucial for carotenoid production. The anaplerotic role of the PP pathway was important to supply precursors for the ED pathway. A balanced metabolic flux distribution among precursor supply, NADPH generation, and by-product pathways was established.

**Electronic supplementary material:**

The online version of this article (doi:10.1186/s12934-015-0301-x) contains supplementary material, which is available to authorized users.

## Background

Bioconversion by engineered microbes in cell factories is a promising alternative strategy to produce valuable chemicals, many of which are expensive to produce in their native hosts or by chemical processes [1, 2]. To construct an engineered strain with optimal phenotypes, the first steps are to enhance native pathways or introduce superior heterogeneous pathways for biosynthesis of target chemicals. Another strategy that can be more important and laborious is to seek and integrate separate genetic modification targets that improve productivity, even though the mechanisms of these targets are sometimes poorly understood.

The heterologous production of carotenoids in *Escherichia coli* is a well-studied example of an engineered microbial cell factory in which there has been coordinated modification of multiple targets [3–5]. Carotenoids are isoprenoid pigments naturally produced by plants, algae, and photosynthetic bacteria. They have attracted much attention for industrial use because of their diverse physiological effects and their potential applications as antioxidants and nutraceuticals [6–8]. Carotenoids are structurally derived from two universal isoprene units; isopentenyl diphosphate (IPP) and dimethylallyl diphosphate (DMAPP). There are two independent pathways for the biosynthesis of IPP and DMAPP: the mevalonate pathway from the precursor acetyl-CoA in eukaryotic and archaea cells, and the methyl-d-erythritol 4-phosphate pathway (MEP) pathway from the precursors pyruvate and glyceraldehyde-3-phosphate (G3P) in prokaryotic cells and plastids in plants. Bioconversion of carotenoids by engineered strain has been developed based on either of these two pathways [9, 10].

In *E. coli*, MEP pathway-dependent carotenoid biosynthesis was initially engineered by introducing the carotenoid synthesis gene cluster *crt*EBI and by enhancing the expressions of genes in the MEP pathway, such as *dxs* and *idi* [9]. The modification of chromosomal targets has been shown to further improve carotenoid production [3, 11, 12]. *Pyk*FA was the first gene target identified to improve carotenoid production. Deletion of *pyk*FA reduced the flux from G3P to pyruvate, suggesting that the balance between G3P and pyruvate was important for carotenoid synthesis [13]. Additional rate-limiting targets for carotenoid biosynthesis were further explored using either of two major methods: systematic approaches using genome-scale modeling; and combinatorial approaches based on phenotypic diversification and screening. The targets included the global regulators Hnr and ClpXP; the central metabolism enzymes YtjC and AceE, which may directly affect precursor levels by catalyzing G3P- and pyruvate-related metabolic reactions; and GdhA and FdhF, which might indirectly contribute to cofactors or flux balance [5, 11, 12, 14]. Thus, as suggested, improving and balancing the precursor supply remains an obstacle for MEP pathway-based carotenoid production.

The redistribution of metabolic flux at the pathway level is an alternative strategy to improve chemical biosynthesis and precursor supply [15]. This strategy is always more complex, because desirable flux distribution is not usually achieved by modifying single genes. The MEP pathway begins with the condensation of G3P and pyruvate in equal amounts. These precursors are primarily supplied by the Embden–Meyerhof–Parnas pathway (EMP pathway). The Entner–Doudoroff pathway (ED pathway), as a variant glycolysis pathway, produces equal amounts of G3P and pyruvate. This superior stoichiometric feature makes the ED pathway a preferable route for precursor supply. The strategy to redistribute central metabolic pathways has been used to improve MEP-dependent carotenoid production by engineered microbes [16–18]. However, the exact flux profile is not well understood. Systematic modification and analysis of gene targets in the EMP, ED, and pentose phosphate (PP) pathways may provide new insights on the flux profile.

In addition to the precursor supply, cofactor generation might be another key factor for the MEP pathway and carotenoid biosynthesis. Cofactors such as NAPDH and ATP are necessary for MEP reactions. Furthermore, the bioconversion efficiency could be affected by the accumulation of by-products such as acetate. Thus, it is necessary to elucidate flux distribution among precursor supply, cofactor generation, and by-product pathways. To this end, we implemented an engineering strategy that combined systematic genetic target modification and central metabolic flux redistribution. Multiple gene targets were either knocked out or overexpressed to redistribute the fluxes of the EMP, ED, and PP pathways. The effects of these modifications on the supply of G3P and pyruvate precursors, redox generation, and the metabolism of by-products were analyzed. These results will provide guidance for further MEP-pathway engineering studies.

## Results

### Analysis of metabolic engineering targets within EMP, PP, and ED pathways

**Fig. 1:**
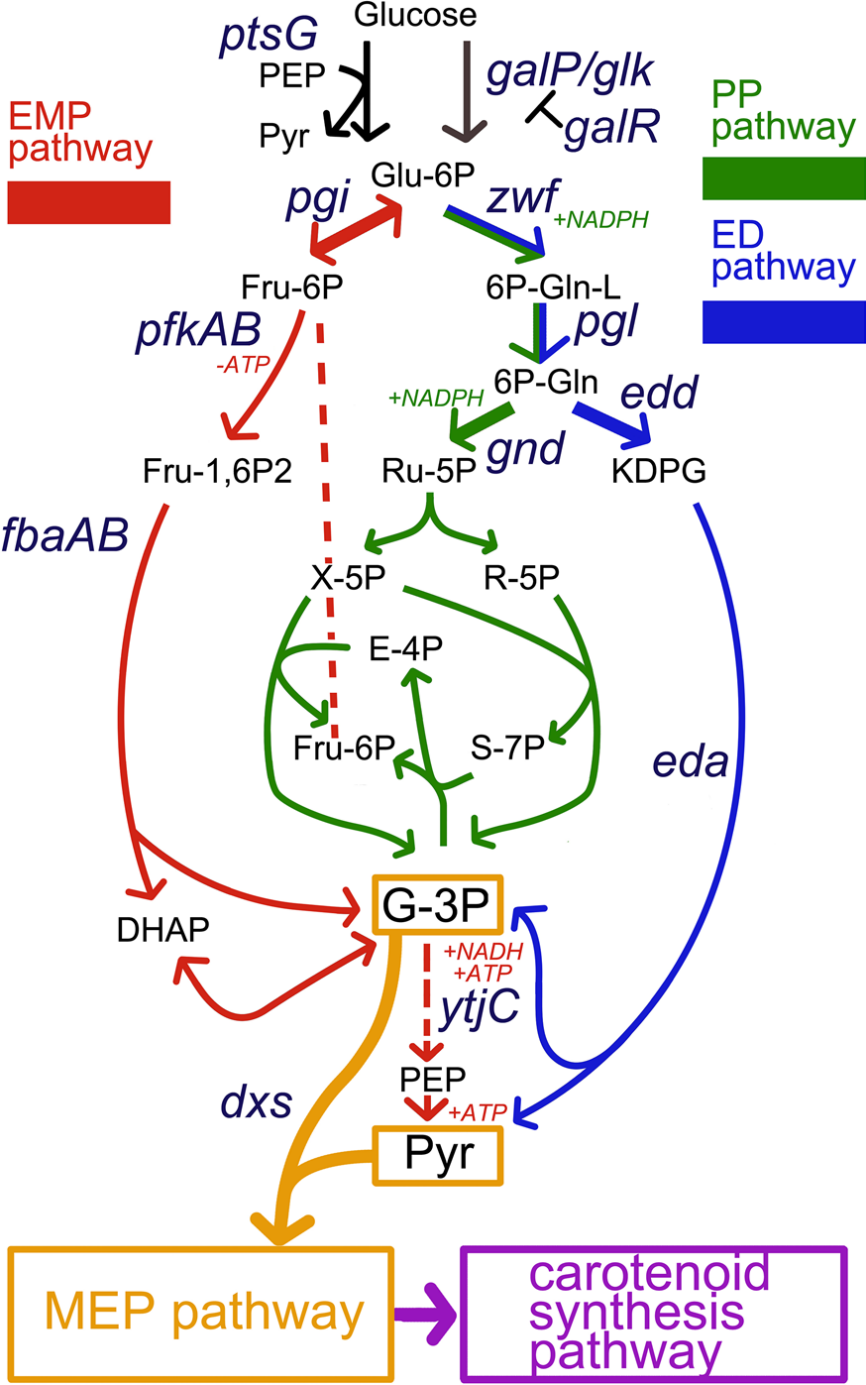
Genetic targets in central metabolic pathways for MEP-dependent carotenoid biosynthesis.

Pyruvate and G3P are the two substrates of 1-deoxy-d-xylulose 5-phosphate synthase (encoded by *dxs*), the first enzyme in the MEP pathway. The favorable stoichiometric ratio of G3P and pyruvate generated via the ED pathway inspired the attempt to develop a non-EMP pathway route for MEP-dependent carotenoid bioconversion. In the EMP pathway, there are three steps in the conversion of the starting compound glucose-6-phosphate (G6P) into G3P, the precursor of the MEP pathway. Phosphoglucose isomerase (encoded by *pgi*) catalyzes the first glycolytic reaction at the branch point of the EMP and ED/PP pathways. The following two reaction steps are catalyzed by 6-phosphofructokinase I/II (encoded by *pfk*A and *pfk*B) and fructose bisphosphate aldolase II/I (encoded by *fba*A and *fba*B), respectively. Theoretically, blocking either of these genes would direct carbon flux from the EMP pathway to the ED/PP pathway, resulting in enhanced flux through the ED pathway. However, deletion of *pgi* disrupted the recycling of F6P or G3P to the oxidative PP pathway, resulting in decreased NADPH generation [19]. In the lower part of the EMP pathway, the conversion from G3P to pyruvate comprises five steps. Among the genes involved in these steps, *ytj*C and *pyk*FA have been identified as targets for modification to improve MEP-dependent biosynthesis. The ED pathway is a shortcut for G3P and pyruvate generation. It produces the two MEP precursors in equal amounts via a four-step reaction from G6P. The ED pathway shares the first two steps with the PP pathway. The key gene targets within the ED and PP pathways are shown in Fig. 1. Compared with the ED pathway, the PP pathway generates one additional molecule of NADPH per molecule of glucose, which might be necessary for a MEP pathway reaction.

### Analysis of targets responsible for directing flux from EMP pathway to ED/PP pathways

Initially, we constructed two reporter vectors for high carotenoid production, pLY036 and pLY10RK; their products were neurosporene and lycopene, respectively. The *crt*EBI cassette alone was insufficient for high carotenoid production (data not shown). Therefore, an additional *E. coliidi* gene was inserted downstream of *crt*EBI. The yields of neurosporene and lycopene from pLY036 and pLY10RK in wild *E. coli* BW25113 were 2.05 and 1.64 mg/g dry cell weight (DCW), respectively.

**Fig. 2:**
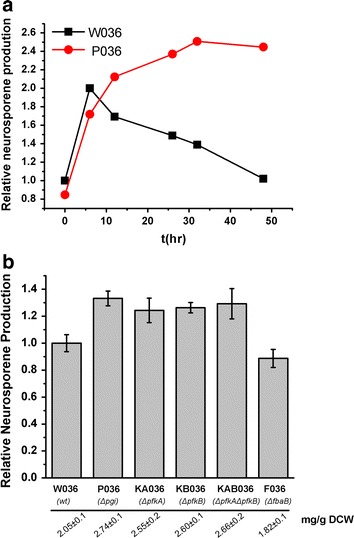
Effects of deleting different EMP pathway genes on neurosporene biosynthesis. **a** Relative neurosporene production of W036 (wild type) and P036 (Δ*pgi*) over time; **b** relative neurosporene production of strains with deletions of different EMP pathway genes after bioconversion for 12 h.

Various target genes within the EMP pathway were deleted to eliminate the flux of G3P and pyruvate through the EMP pathway, and the effects on neurosporene production were investigated. First, neurosporene production over time was compared between the Δ*pgi* strain (P036) and the wild-type strain (W036) (Fig. 2a). The neurosporene yield of P036 remained stable during cell culture and was consistently greater than that of W036. The highest yield of P036 (after cell culture for 30 h) was approximately 25% greater than that of W036. The W036 strain produced the highest yield at the beginning of bioconversion, and then the yield gradually decreased. Considering cell growth and turnover, the decrease in neurosporene production per cell mass in W036 might be because neurosporene did not further accumulate during bioconversion. In contrast, the deletion of *pgi* might have resulted in the continued supply of optimal precursors or cofactors for the MEP pathway. The effects of deletions of other EMP genes were then investigated (Fig. 2b, biomass and carotenoids production of all strains in this study are summarized in Additional file 1: Table S1). Compared with the wild-type strain, the Δ*pfk*A and Δ*pfkB* strains (KA036 and KB036) and the Δ*pfk*AΔ*pfk*B strain (KAB036) produced approximately 25% more neurosporene, similar to P036. As for the targets of fructose bisphosphate aldolase, only *fba*B was a non-lethal deletion. However, deletion of *fba*B (strain F036) had no effect on neurosporene production.

**Fig. 3:**
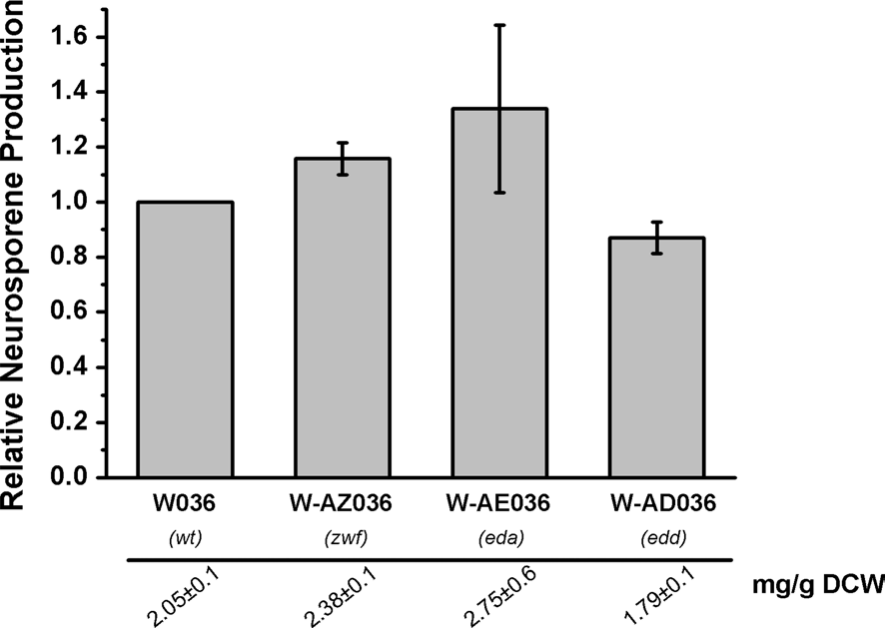
Effects of overexpression of different ED/PP pathway genes on neurosporene biosynthesis. Relative neurosporene production of strains overexpressing different ED/PP pathway genes after bioconversion for 12 h.

To further investigate the effects of central flux redistribution on carotenoid biosynthesis, three branching point enzymes within the PP and ED pathways, *zwf*, *edd*, and *eda*, were each overexpressed in *E. coli*, and neurosporene production was compared among the overexpressing strains. As shown in Fig. 3, overexpression of Zwf (strain Z036) and Eda (strain E036) increased neurosporene yields by approximately 20 and 12%, respectively, while overexpression of *edd* in strain D036 decreased the yield by approximately 20%. The SDS-PAGE results (see Additional file 2: Figure S1) implied that *edd* overexpression might interfere with the normal expression of other genes such as *crt*I, resulting in decreased neurosporene production. These findings indicate that neurosporene biosynthesis could be improved by engineering target genes that could redistribute flux towards the ED/PP pathways, especially the ED pathway.

### Efficient substrate use of G3P and pyruvate by overexpression of *dxs* in ED/PP pathways

**Fig. 4:**
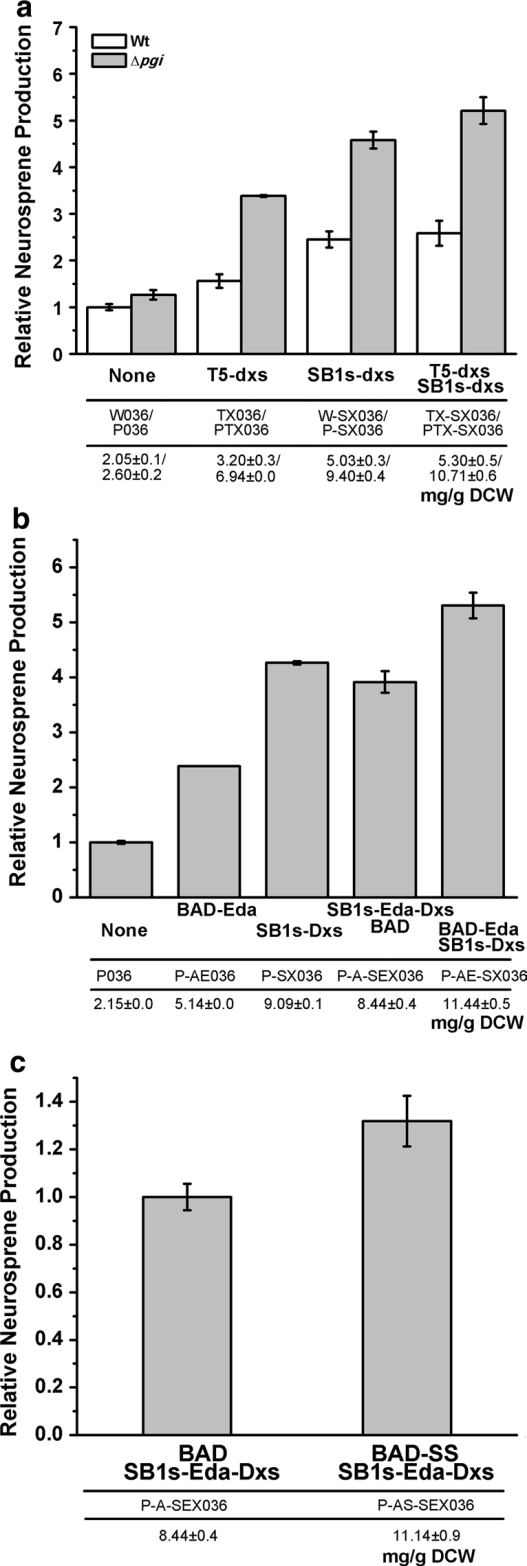
Enhanced precursor use by manipulating Dxs and Eda. **a** Dxs was expressed at different strengths, and neurosporene production was compared between Δ*pgi* strain and wild-type strain after bioconversion for 12 h; **b** neurosporene production in strain simultaneously expressing Dxs and Eda after bioconversion for 12 h; **c** neurosporene production in strain in which Dxs and Eda were spatially coupled by protein scaffold after bioconversion for 12 h.

To further study the effects of redistributed metabolic routes on carotenoid production, the pathways were modified in a MEP pathway-enhanced background. The first enzyme of the MEP pathway, Dxs, was overexpressed to increase the flux into the MEP pathway. This was achieved using three strategies: replacing the native promoter of the chromosomal *dxs* gene with the strong constitutive T5 promoter, which produced 3.20 mg/g DCW neurosporene (strain TX036); overexpressing the *E. colidxs* gene in a low copy-number plasmid (pSC101) under the control of the *ara*BAD promoter (pSB1s-*dxs*), resulting in 5.03 mg/g DCW neurosporene production in the wild-type strain (SX036); and combining these two strategies, resulting in a neurosporene yield of 5.30 mg/g DCW (TX-SX036). These three strategies were also applied to the Δ*pgi* strain (PTX036 for chromosomal T5 promoter *dxs* strain, P-SX036 for the plasmid *dxs* strain, PTX-SX036 for the combined strategy strain) and neurosporene production was compared with that of the wild-type strain. After bioconversion, the neurosporene yields of PTX036, P-SX036, and PTX-SX036 were 6.94, 9.40, and 10.71 mg/g DCW respectively, about 1.51–1.88 times more than that of the wild-type strain (Fig. 4a). This result implied that the substrate use of Dxs was significantly improved, possibly because of a balanced G3P and pyruvate supply from the ED pathway.

To further verify the role of the ED pathway, we attempted to improve the precursor supply by coordinated improvements to Eda and Dxs. First, we overexpressed Eda and Dxs simultaneously in the Δ*pgi* strain. Eda and Dxs were expressed in the pBAD and pSB1s plasmids (P-AE036 and P-SX036, and co-transformation of the two plasmids for P-AE-SX036), respectively. As shown in Fig. 4b, the production of neurosporene was increased 25% by Eda enhancement, as compared with those of P-SX036 and P-AE-SX036. Second, a protein scaffold was used to spatially recruit Eda and Dxs through the interaction between tagged ligands. We designed a protein scaffold platform based on two proteins from *Clostridium* sp.; cellulosome cohesin and dockerin. Eda and Dxs were each fused with different dockerins and were co-expressed with scaffold protein (SS) containing a cohesin domain in the Δ*pgi* strain. The function of SS was confirmed in a His-tag pull down assay (Additional file 3: Figure S2). Eda and Dxs co-localized with each other via the interaction between dockerins and SS. To balance the expression level with the scaffold, Eda and Dxs were co-transcribed under the control of the *ara*BAD promoter using the low-copy pSB1s plasmid in the Δ*pgi* strain (P-A-SEX036), compatible with SS expression using the pBAD plasmid. As shown in Fig. 4c, neurosporene production was increased by approximately 30% in the scaffold platform strain (P-AS-SEX036). These results confirmed the significant role of the ED pathway in carotenoid production.

### Fine-tuning *gnd* expression to balance PP and ED pathway flux

**Fig. 5:**
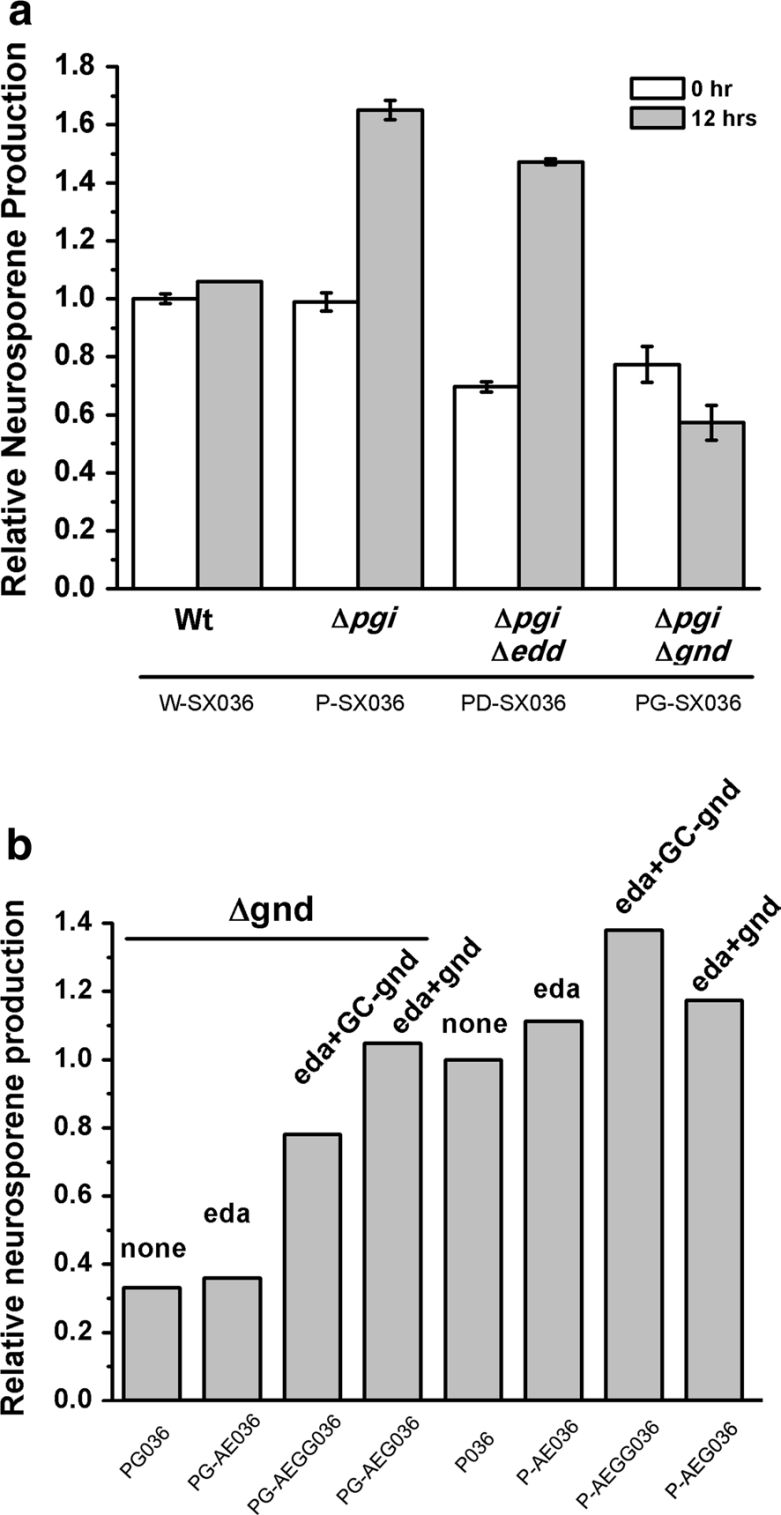
Roles of PP pathway and ED pathway in ED/PP pathway route and balance between PP and ED pathways. **a** Neurosporene production in strains Δ*pgi*Δ*edd* and Δ*pgi*Δ*gnd* after bioconversion for 0 or 12 h; **b** neurosporene production in strains with different *gnd* and *eda* engineering genotypes after bioconversion for 12 h.

The above results indicated that modification of ED targets resulted in more efficient precursor use by the MEP pathway. However, the ED pathway does not generate sufficient NADPH for MEP reactions. To explore the balance between the ED and PP pathways in this metabolic engineering route, either *edd* or *gnd* was knocked out in the Δ*pgi* strain to construct PP-only and ED-only strains. The MEP pathway was enhanced by introducing pSB1s-*dxs*. In the bioconversion experiments, neurosporene production was significantly decreased in both PP-only (PD-SX036) and ED-only (PG-SX036) strains (Fig. 5a), indicating that both the PP pathway and the ED pathway were important for MEP-dependent biosynthesis. Thus, fine-tuning the flux between the ED pathway and the PP pathway was necessary to optimize the bioconversion process.

Different flux distributions between the PP and ED pathways were achieved by coordinated engineering of *gnd* and *eda* expression. In this experiment, *gnd* was overexpressed at two different levels in either the wild-type or the Δ*gnd* background: high-level expression was obtained using the *ara*BAD promoter; low-level expression was obtained by introducing rare codons (Gly-Pro) at the N-terminal of Gnd peptides under the control of the *ara*BAD promoter (Additional file 4: Figure S3). *gnd* was co-expressed with *eda*. The results indicated that moderate *gnd* expression combined with *eda* was most favorable for neurosporene synthesis in the wild-type background (Fig. 5b).

### Anaplerotic role of PP pathway is important for carotenoid biosynthesis

**Fig. 6:**
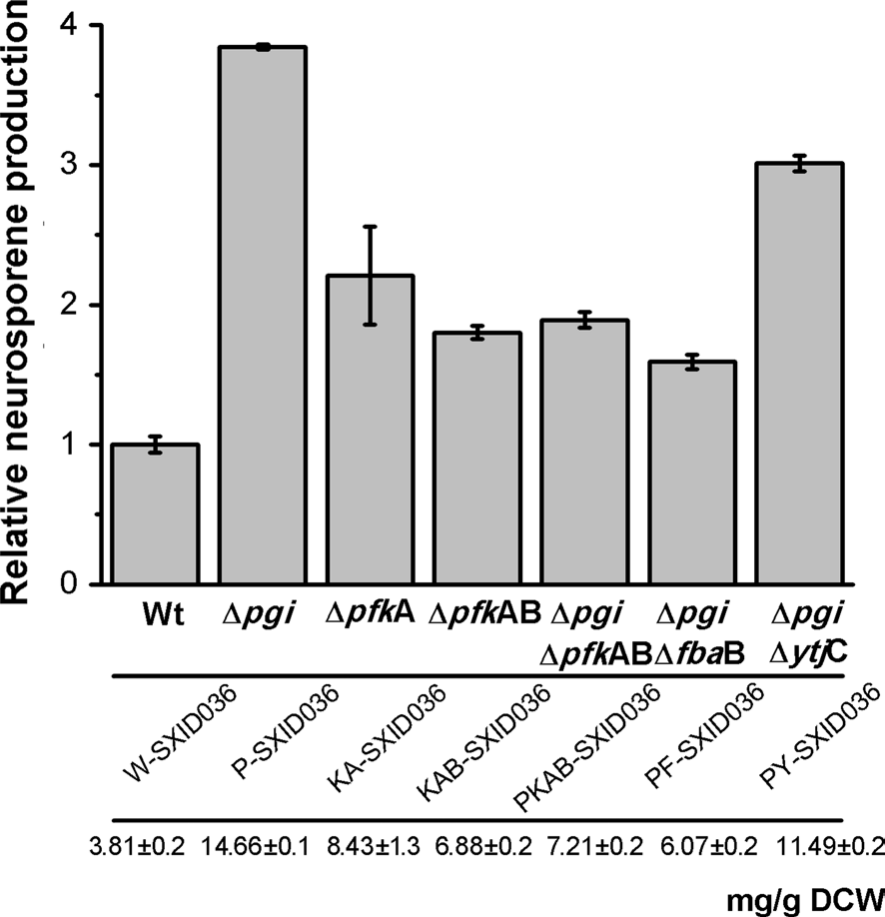
Flux balance within EMP pathway and its effects on carotenoid biosynthesis. Neurosporene production in strains with deletions of different EMP pathway targets after bioconversion for 12 h.

Next, different targets were modified to determine the effects of PP pathway on neurosporene production. Compared with Δ*pgi*, Δ*pfk*AB generated more NADPH through partial cyclization of the PP pathway [19]. Δ*pfk*A or Δ*pfk*AB have been reported as useful targets to improve NAPDH generation for chemical production [20, 21]. If the PP pathway contributes to neurosporene synthesis mainly by generating NAPDH, then further improvement of the NADPH supply will benefit neurosporene synthesis. To test this assumption, Δ*pgi* was replaced by Δ*pfk*A or Δ*pfk*AB in an MEP-enhanced background. A novel plasmid pSB1s-XID was used to further improve the MEP flux, in which *E. coliidi* and *isp*DF were located downstream of *dxs* in pSB1s-*dxs*. The neurosporene yields of both KA-SXID036 (Δ*pfk*A) and KAB-SXID036 (Δ*pfk*AB) were lower than that of P-SXID036 (Δ*pgi*) (Fig. 6). We suspected that the PP pathway might play additional roles other than simply generating NADPH for the ED pathway. We further deleted the lower EMP pathway targets, *pfk*A, *pfk*AB, *fba*B, and *ytj*C in the Δ*pgi* background, aiming to block the anaplerotic flux from the PP pathway towards G3P and pyruvate. Neurosporene production was decreased in all these strains (Fig. 6). Together, these results suggested that the PP pathway might play an anaplerotic role in supplying precursors for the ED pathway.

### Enhanced carotenoid biosynthesis is accompanied with improved biomass and decreased acetate overflow

**Fig. 7:**
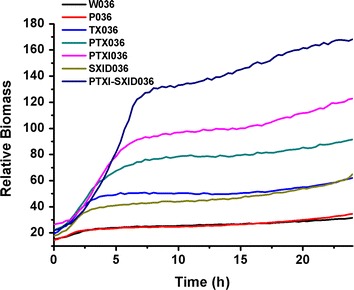
Batch fermentation to produce neurosporene in a microbioreactor. Change in relative biomass over time for strains W036 (BW25113 with pLY036), P036 (*Δpgi* with pLY036), TX036 (P_T5_-*dxs* with pLY036), PTX036 (*Δpgi* P_T5_-*dxs* with pLY036), PTXI036 (*Δpgi* P_T5_-*dxs* P_T5_-*idi* with pLY036), SXID036 (BW25113 with pSB1s-*dxs*-*idi*-*isp*DF and pLY036), PTXI-SXID036 (*Δpgi* P_T5_-*dxs* P_T5_-*idi* with pSB1s-*dxs*-*idi*-*isp*DF and pLY036).

Redistribution of central metabolic flux always has complex effects. To investigate the balance between carotenoid biosynthesis and other cell metabolic pathways, batch fermentation for neurosporene production was conducted in a microbioreactor. As the MEP pathway flux still played a significant role in carotenoid production, the MEP pathway was further improved by replacing the chromosomal promoter with the T5 promoter to drive *idi*, resulting in PTXI036 and PTXI-SXID036 (containing pSB1s-*dxs*-*idi*-*isp*DF). Both biomass and neurosporene production were monitored in these strains. Surprisingly, the synthesis of neurosporene was beneficial for cell growth in the ED/PP pathway strains but not in the wild-type strain. As shown in Fig. 7 and Table 1, the strains with greater neurosporene synthesis showed greater cell growth and biomass, except for SXID036, which did not have modified central metabolic pathways. Thus, the inclusion of ED/PP pathways led to significant improvements in the total carotenoid titer.

**Table 1:** Batch fermentation for neurosporene production in a microbioreactor

Strain	Biomass (OD_600_ value)	Neurosporene production (mg neurosporene/g DCW)	Titer (mg neurosporene/L)
W036	8.91	1.76	4.7
P036	10.35	1.83	5.68
TX036	16.2	2.40	11.66
PTX036	23.16	4.64	32.24
PTXI036	30.35	5.35	48.71
W-SXID036	17.3	4.53	23.51
PTXI-SXID036	40.47	16.69	202.64

Bioconversion was performed in a microbioreactor for 24 h and biomass and neurosporene production were monitored.

Next, we analyzed metabolic intermediates to elucidate the coupling mechanism between biomass accumulation and carotenoid biosynthesis. The strains were cultured in flasks, and acetate accumulation and glucose consumption were monitored. The biomass results were similar to those observed in the microbioreactor experiments. Improvement of the MEP pathway in the Δ*pgi* background resulted in significantly reduced acetate accumulation and enhanced glucose consumption (Table 2). Thus, we proposed that efficient use of glucose in the MEP pathway can establish a flux distribution that is favorable not only for carotenoid production but also for cell growth. This is probably because of the blockage of some by-product pathways, such as acetate overflow.

**Table 2:** Acetate accumulation and glucose consumption of strains

Strain	Biomass (OD_600_)	Acetate production (mg acetate/mL)	Residual glucose (mg glucose/mL)
W036	10	7.86	14.6
P036	10.8	5.80	7.8
W-SXID036	13.1	2.28	0.03
PSX036	18.3	0.49	0
PTXI-SXID036	22.6	0.21	0

Strains were cultured in flasks for bioconversion, and acetate accumulation and glucose consumption were monitored.

### Combined modification of targets in ED/PP pathways for efficient lycopene production

**Fig. 8:**
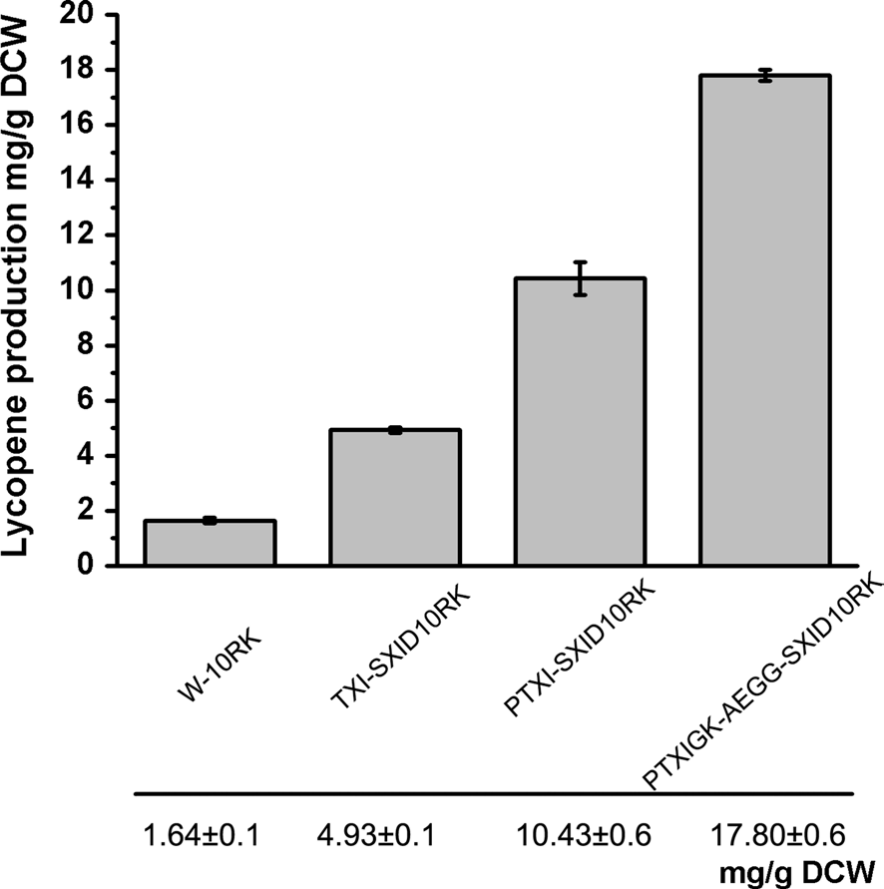
Lycopene production in strains with ED/PP pathway route. Lycopene production in wild-type strain (W-10RK, BW25113 with pLY10RK), non-ED/PP route strain (TXI-SXID10RK, P_T5_-*dxs* P_T5_-*idi with* pSB1s-*dx* -*idi*-*isp*DF and pLY10RK), ED/PP route strain (PTXI-SXID10RK, *Δpgi* P_T5_-*dxs* P_T5_-*idi with* pSB1s-*dx* -*idi*-*isp*DF and pLY10RK), and final optimal engineered strain (PTXIGK-AEGG-SXID10RK, *Δpgi*P_T5_-*dxs*P_T5_-*idi*Δ*pts*G*gal*R::P_119_-*glk* with pBAD-*eda*-*GCgnd*, pSB1s-*dxs*-*idi*-*isp*DF and pLY10RK) after bioconversion for 24 h.

Finally, we developed an optimally engineered strain based on the ED/PP pathway route (based on Δ*pgi*, T5-*dxs*-*idi*, pBAD-*eda*-GC*gnd,* and pSB1s-*dxs*-*idi*-*isp*DF) for lycopene production using the reporter vector pLY10RK. To improve the total flux for lycopene production, glucose supply was optimized by engineering a glucose-uptake pathway. Namely, *pts*G (encoding glucose PTS permease) was knocked out to reduce consumption of pyruvate and ATP. The non-phosphotransferase system was improved by inserting the P119-*glk* cassette (glucokinase gene *glk* under the control of the strong constitutive promoter P119) at the chromosomal *gal*R locus [releasing the repression of the galactose repressor GalR to increase expression of galactose permease (GalP)]. Combining these targets, the resulting strain (PTXIGK-AEGG-SXID10RK) produced 17.8 ± 0.2 mg lycopene/g DCW in flask culture (Fig. 8). The lycopene production was about 3.6 times higher than that of the non ED/PP pathway route strain TXI-SXID10RK.

## Discussion and conclusions

An ideal microbial cell factory for bioconversion should maximize the target metabolic flux with an exact stoichiometric ratio of precursors and cofactors. However, this is hard to achieve because of the complexity of metabolic flux and its regulation. For MEP-dependent carotenoid biosynthesis, imbalanced precursor supply is one of the major metabolic bottlenecks, especially considering the limit of precursor (G3P) availability. Disruption of the EMP pathway target *pyk*FA has been shown enhance lycopene production by retarding the catabolism of G3P to pyruvate [13]. In another study, the unexpected accumulation of indole may indicate that the pool of the G3P precursor was insufficient, because indole may be a by-product of an anaplerotic supply of G3P through the tryptophan biosynthesis pathway during MEP-dependent taxol production [22]. Research on non-EMP pathway precursor supply routes has mainly focused on the deletion of *pgi* [16, 18]. However, a parallel study has shown that lycopene yield was improved in a reverse redistribution pathway route with knocked-out *zwf* [17]. This indicated that detailed studies of both flux and targets were necessary. Herein, we conducted a systematic analysis and modification of genetic targets within central metabolic pathways for the redistribution of metabolic flux for MEP-dependent carotenoid biosynthesis.

**Fig. 9:**
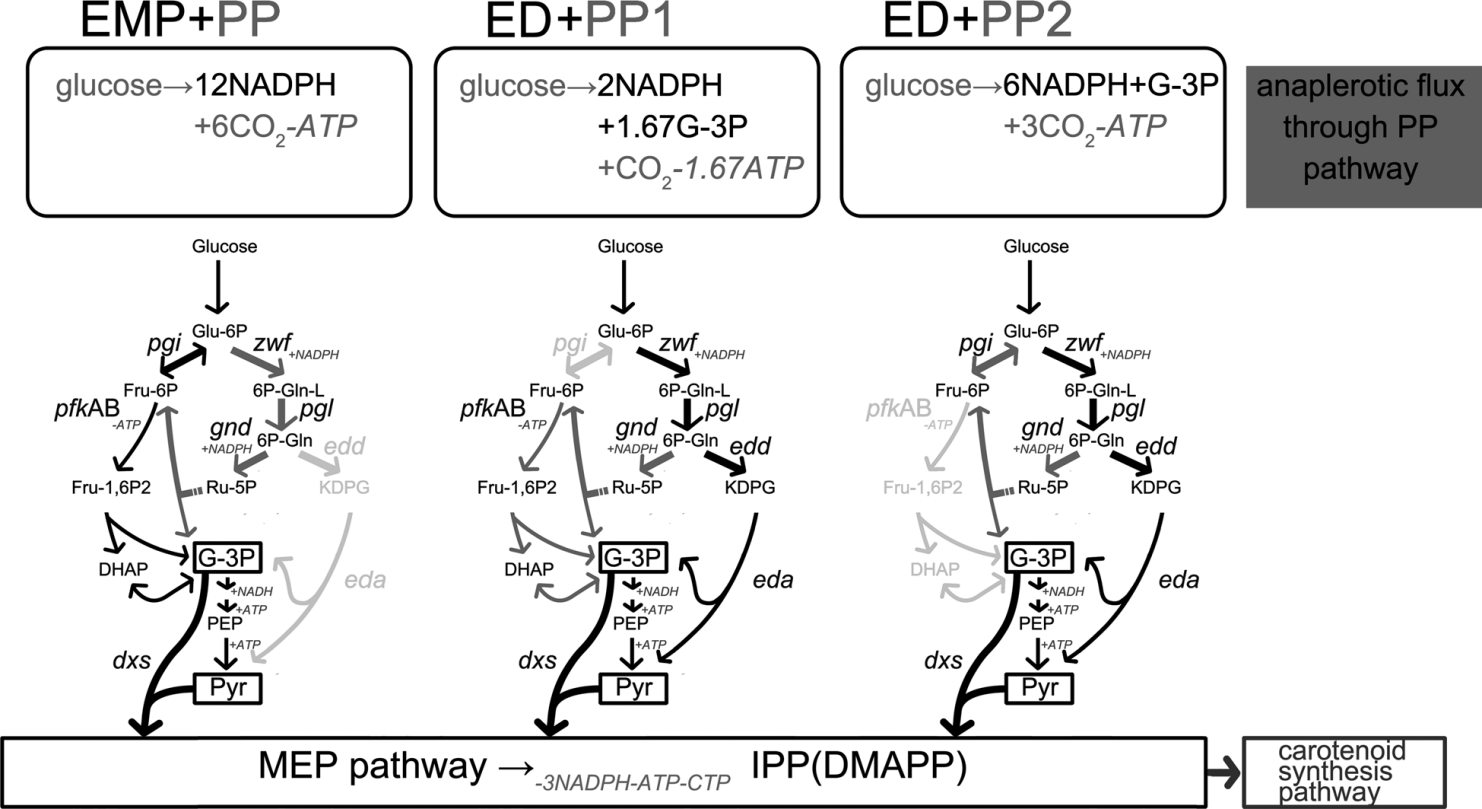
NADPH and G3P generation through different pathway routes. ED/PP pathways routes were created by either Δ*pgi* or Δ*pfk*AB, resulting in ED + PP1 or ED + PP2. EMP or ED pathway is shown in* black*; PP pathway is shown in *dark gray*. NADPH and G3P generation through PP pathways in different routes is summarized in corresponding *boxes.*

As expected, the ED/PP pathway route created by deleting EMP targets and/or improving ED/PP targets enhanced carotenoid biosynthesis. Eda and Dxs seemed to be key targets for improving carotenoid biosynthesis. This was most likely because of their roles in the balance and efficient use of G3P and pyruvate precursors supplied by the ED pathway. Further metabolic engineering work will focus on coordinated modifications of Eda and Dxs. The flux balance between the ED and PP pathways was optimized by fine tuning *gnd* expression. Weak *gnd* expression was better for carotenoid synthesis, indicating the importance of the proper flux ratio between the ED and PP pathways. Although the ED pathway generated precursors with an exactly balanced ratio for MEP-dependent biosynthesis, the precursor balance still might be perturbed as both G3P and pyruvate could be metabolized in other cellular pathways. The results of this study implied that the anaplerotic role of the PP pathway might be important for precursor supply. The first clue was that the lower part of the MEP pathway was still necessary for ED/PP pathway route-dependent carotenoid biosynthesis. In addition, the carotenoid yields of Δ*pgi* strains were greater than those of Δ*pfk*AB strains. The PP pathway routes achieved by Δ*pgi* and Δ*pfk*AB might differ in their theoretical flux distribution [21]. Deletion of *pgi* would block the recycling of F6P and G3P into the oxidative PP pathway, and increase flux into the lower part of the EMP pathway (PP1, Fig. 9). However, *pfk*AB deletion might create a partial cyclization for the recovery of F6P from the PP pathway (PP2, Fig. 9). More NADPH was generated by the PP2 route than by the PP1 route, while the PP1 route supplied more G3P (Fig. 9). A pathway route based on Δ*pgi* (ED + PP1) was more favorable for MEP-dependent biosynthesis. This suggested that the major role of the PP pathway was to sustain the G3P pool, although NADPH generated by the PP pathway was also important because the ED pathway was redox imbalanced for MEP-pathway reactions. However, detailed flux and metabolite analyses are required to determine the exact metabolic profile.

The bioconversion efficiency of a given chemical is determined by both the stoichiometric yield of the pathway and the kinetic efficiency of pathway reactions. The metabolic route established by deleting *pgi* not only provided a balanced precursor ratio for efficient turnover in the MEP pathway, but also created a well-distributed metabolic flux between carotenoid biosynthesis and other pathways. Acetate overflow is always a major metabolic burden in *E. coli* K-12 strains because of the imbalance of central metabolic flux in high-glucose culture conditions. Surprisingly, the improvement of carotenoid biosynthesis in the redistribution route led to both greater cell growth and less acetate accumulation. This indicated that MEP-dependent carotenoid biosynthesis might relieve the metabolic burden by balancing the level of redox or metabolites, e.g., pyruvate. Indeed, redistribution of central metabolic flux always has complex effects. A global flux response could occur when MEP precursors are used efficiently or when redox conditions are balanced, resulting in flexible adaptation of flux distribution. Additional factors in central metabolic pathways, such as the level of the regulator intermediate fructose 6-bisphosphate, might be affected in the redistribution route, resulting in various responses and effects on the carotenoid biosynthesis pathway. Further research should focus on the effects of global regulation of central metabolic networks and their flux in carotenoid biosynthesis.

## Methods

### Bacterial strains and plasmids

*Escherichia coli* BW25113 was used as the parental strain for genetic modification and carotenoid production. Gene knock-out strains were obtained from the KEIO collection [23]. *Escherichia coli* DH5α was used for standard plasmid construction. The plasmids used for MEP gene expression and as reporter vectors were derived from expression vectors previously developed in our laboratory (unpublished). These vectors have the following features: a promoter (*ara*BAD), multiple cloning sites, the rrnB terminator, origin of replication (p15A, RSF1030 or pSC101), and an antibiotic resistance gene (Kan, Cm, or Sm resistance). The plasmids pUKM (derivative of pKD4), pKD46, and pCP20 were used for gene integration. All *E. coli* strains and plasmids used in this study are described in Table 3.

**Table 3:** Strains and plasmids used in this study

Strain/plasmid	Description	References
*Escherichia coli* strains		
BW25113	Wild type K-12 strain	Our lab
DH5αa	For genetic manipulation	Invitrogen
TX	P_T5_-*dxs* K-12 strain, for construction of strains that harboring P_T5_-*dxs* phenotype	This study
TI	P_T5_-*idi* K-12 strain, for construction of strains that harboring P_T5_-*idi* phenotype	This study
GRK	*gal*R::P_119_-*glk* K-12 strain, for construction of strains that harboring *gal*R::P_119_-*glk* phenotype	This study
W036	BW25113, pLY036 plasmid	This study
P036	*Δpgi*, pLY036 plasmid	This study, KEIO
KA036	*Δpfk*A, pLY036 plasmid	This study, KEIO
KB036	*Δpfk*B, pLY036 plasmid	This study, KEIO
KAB036	*Δpfk*A*Δpfk*B, pLY036 plasmid	This study
F036	*Δfba*B, pLY036 plasmid	This study, KEIO
W-AZ036	BW25113, pBAD-*zwf* and pLY036 plasmids	This study
W-AE036	BW25113, pBAD-*eda* and pLY036 plasmids	This study
W-AD036	BW25113, pBAD-*edd* and pLY036 plasmids	This study
TX036	P_T5_-*dxs*, pLY036 plasmid	This study
PTX036	*Δpgi*P_T5_-*dxs*, pLY036 plasmid	This study
W-SX036	BW25113, pSB1s-*dxs* and pLY036 plasmids	This study
P-SX036	*Δpgi*, pSB1s-*dxs* and pLY036 plasmids	This study, KEIO
TX-SX036	P_T5_-*dxs*, pSB1s-*dxs* and pLY036 plasmids	This study
PTX-SX036	*Δpgi* P_T5_-*dxs*, pSB1s-*dxs* and pLY036 plasmids	This study
P-AE036	*Δpgi*, pBAD-*eda* and pLY036 plasmids	This study, KEIO
P-A-SEX036	*Δpgi*, pBAD, pSB1s-*eda*-*dxs* and pLY036 plasmids	This study, KEIO
P-AE-SX036	*Δpgi*, pBAD-*eda*, pSB1s- *dxs* and pLY036 plasmids	This study, KEIO
P-AS-SEX036	*Δpgi*, pBAD-SS, pSB1s-*eda*-*dxs* and pLY036 plasmids	This study, KEIO
PD-SX036	*ΔpgiΔedd*, pSB1s-*dxs* and pLY036 plasmids	This study
PG-SX036	*ΔpgiΔgnd*, pSB1s-*dxs* and pLY036 plasmids	This study
P-AEG036	*Δpgi*, pBAD-*eda*-*gnd* and pLY036 plasmids	This study, KEIO
P-AEGG036	*Δpgi*, pBAD-*eda*-*GCgnd* and pLY036 plasmids	This study, KEIO
PG036	*ΔpgiΔgnd*, pLY036 plasmid	This study
PG-AE036	*ΔpgiΔgnd*, pBAD-*eda* and pLY036 plasmids	This study
PG-AEG036	*ΔpgiΔgnd*, pBAD-*eda*-*gnd* and pLY036 plasmids	This study
PG-AEGG036	*ΔpgiΔgnd*, pBAD-*eda*-*GCgnd* and pLY036 plasmids	This study
W-SXID036	BW25113, pSB1s-*dxs*-*idi*-*isp*DF and pLY036 plasmids	This study
P-SXID036	*Δpgi*, pSB1s-*dxs*-*idi*-*isp*DF and pLY036 plasmids	This study, KEIO
KA-SXID036	*Δpfk*A, pSB1s-*dxs*-*idi*-*isp*DF and pLY036 plasmids	This study
KAB-SXID036	*Δpfk*A*Δpfk*B, pSB1s-*dxs*-*idi*-*isp*DF and pLY036 plasmids	This study
PKAB-SXID036	*ΔpgiΔpfk*A*Δpfk*B, pSB1s-*dxs*-*idi*-*isp*DF and pLY036 plasmids	This study
PF-SXID036	*ΔpgiΔfba*B, pSB1s-*dxs*-*idi*-*isp*DF and pLY036 plasmids	This study
PY-SXID036	*ΔpgiΔytj*C, pSB1s-*dxs*-*idi*-*isp*DF and pLY036 plasmids	This study
PTXI036	*Δpgi* P_T5_-*dxs* P_T5_-*idi*, pLY036 plasmids	This study
PTXI-SXID036	*Δpgi* P_T5_-*dxs* P_T5_-*idi*, pSB1s-*dx* -*idi*-*isp*DF and pLY036 plasmids	This study
W10RK	BW25113, pLY10RK plasmid	This study
TXI-SXID10RK	P_T5_-*dxs* P_T5_-*idi*, pSB1s-*dx* -*idi*-*isp*DF and pLY10RK plasmids	This study
PTXI-SXID10RK	*Δpgi* P_T5_-*dxs* P_T5_-*idi*, pSB1s-*dx* -*idi*-*isp*DF and pLY10RK plasmids	This study, KEIO
PTXIGK-AEGG-SXID10RK	*Δpgi,* P_T5_-*dxs*P_T5_-*idi*, Δ*pts*G*gal*R::P_119_-*glk,* pBAD-*eda*-*GCgnd*, pSB1s-*dxs*-*idi*-*isp*DF and pLY10RK plasmids	This study, KEIO
Other bacterial strains		
*Anabaena sp.* PCC7120	*crt*E, *crt*B for pLY036	Our lab
*Rhodobacter sphaeroides* 2.4.1	*crt*I for pLY036	Our lab
*Pantoea ananatis*	*crt*B. *crt*I for pLY10RK	Our lab
*Pantoea agglomerans*	*crt*E for pLY10RK	Our lab
Plasmids		
pKD46	For lambda-Red mediated recombination	[25]
pUKM	For lambda-Red mediated recombination, derived from pKD4 by inserting multiple cloning site closed to FRT	Our lab
pCP20	For lambda-Red mediated recombination	[25]
pUKM-T5	For construction of TX and TI, derived from pUKM by inserting T5 promoter at multiple cloning site	This study
pBAD-HisA	ColE1 origin, *ara*BAD promoter, Amp^R^	Invitrogen
pSB1s	pSC101 origin, *ara*BAD promoter, Str^R^	Our lab
pS95s	pSC101 origin, constitutive P119 promoter (derived from iGEM part BBa_J23119), Str^R^	Our lab
pS95s-*glk*	pSC101 origin, constitutive P119 promoter, Str^R^,*E. coli glk*, for construction of pUKM-*glk*	This study
pUKM-*glk*	For construction of GRK, derived from pUKM by inserting P_119_-*glk* fragment at multiple cloning site	This study
pLY036	p15A origin, *ara*BAD promoter, Cm^R^, *Anabaena sp crt*EB, *R. sphaeroides crt*I*, E. coli idi*	This study
pLY10RK	RSF1030 origin, *ara*BAD promoter, Kan^R^, *P*. *agglomerans crt*E, *P. ananatis crt*BI*, E. coli idi*	This study
pSB1s-*dxs*	pSC101 origin, *ara*BAD promoter, Str^R^,*E. coli dxs*	This study
pSB1s-*eda*-*dxs*	pSC101 origin, *ara*BAD promoter, Str^R^, dockerin-fused *E. coli eda,* dockerin-fused *E. coli dxs*	This study
pSB1s-*dxs*-*idi*-*isp*DF	pSC101 origin, *ara*BAD promoter, Str^R^, *E. coli dxs,idi,isp*DF	This study
pBAD-*zwf*	ColE1 origin, *ara*BAD promoter, Amp^R^, *E. coli zwf*	This study
pBAD-*edd*	ColE1 origin, *ara*BAD promoter, Amp^R^, *E. coli edd*	This study
pBAD-*eda*	ColE1 origin, *ara*BAD promoter, Amp^R^, *E. coli eda*	This study
pBAD-*eda*-*gnd*	ColE1 origin, *ara*BAD promoter, Amp^R^, *E. coli eda,gnd*	This study
pBAD-*eda*-*GCgnd*	ColE1 origin, *ara*BAD promoter, Amp^R,^ *E. coli eda,GCtag*-*gnd*	This study
pBAD-SS	ColE1 origin, *ara*BAD promoter, Amp^R^, *clostridium sp* cellulose scaffold protein	This study

### Construction of plasmids and genomic integration

Plasmids were constructed using standard molecular biological protocols [24]. For chromosomal promoter replacement and gene integration, heterogeneous gene fragments were inserted at the MCS site downstream of the FRT-kanamycin resistance cassette in pUKM. The gene-FRT-kan-FRT fragment was then amplified by PCR. Lambda-Red-mediated recombination was performed as described elsewhere [25]. For chromosomal phenotype integration, P1 virus-mediated transfection was performed as described elsewhere [26]. The primers used in this study are listed in Additional file 5: Table S2.

### Growth media

LB medium was used for all molecular construction experiments and strain cultures. Auto-inducing medium [27] was used to induce protein expression. Bioconversion medium was prepared by adding 4% (w/v) glucose to M9 medium or LB medium.

### Culture conditions

All strains were stored at −80°C until use. Strains were pre-cultured in LB medium supplemented with appropriate antibiotics at 37°C overnight and then transferred into auto-inducing medium to induce expression of recombinant proteins. For neurosporene and lycopene production, cells were harvested by centrifugation after protein induction. Cells were then transferred to bioconversion medium at a starting biomass of 4 OD/L. For bioconversion experiments, cells were cultured in a flask at 37°C with shaking at 220 rpm for indicated times, and then harvested to quantify products and metabolites.

### Measurement of carotenoid production and production of other metabolites

Cells were harvested by centrifugation at 12,000*g* for 5 min. The cell pellet was washed and then extracted in 1 mL acetone at 4°C for 1 h. The mixture was centrifuged at 12,000*g*, and the absorbance of the supernatant was measured at 470 nm. Neurosporene was quantified as described elsewhere [28]. Lycopene was quantified using a standard curve of pure standard lycopene (Sigma). The biomass was determined by measuring cell density (OD_600_). The acetate concentration was determined by HPLC using a C_18_ column (Agilent), and the glucose concentration was measured using a glucose monitor (SDBI).

### Batch fermentation in a microbioreactor

For batch fermentation of carotenoids, the strains were pre-cultured in auto-inducing medium to induce the synthesis of recombinant proteins. Then, cells were transferred into bioconversion medium in a bioreactor (BioLector), with a beginning cell density of 4 OD. The cells were cultured at 37°C with shaking at 800 rpm.

## Additional files

Additional file 1:
**Table S1.** Biomass and carotenoid production of strains. Additional file 2:
**Figure S1.** SDS-PAGE analysis of recombinant proteins produced by *Escherichia coli* strains: (M) protein markers; (0) strain W036; (1) strain W-AZ036; (2) strain W-AE036; (3) strain W-AD036. Additional file 3:
**Figure S2.** Results of His-tag pull-down assay. Briefly, strains were induced to express target genes, then cells were lysed by sonication. Cell lysates were centrifuged and supernatants were incubated with Ni–NTA beads for 6 h. Beads were washed to remove unbound proteins. Bound proteins were eluted and analyzed by SDS-PAGE. (M) Markers; (1) strain with pSB1s-*eda*-*dxs* (dockerin-fused); (2) strain with pBAD-SS (His-tag fused scaffold protein); (3) strain with pBAD-SS and pSB1s-*dxs* (dockerin-fused); (4) strain with pBAD-SS and pSB1s-*eda*-*dxs*. Additional file 4:
**Figure S3.** SDS-PAGE analysis of recombinant proteins produced by *Escherichia coli* strains: (M) protein markers; (1) strain P036; (2) strain P-AE036; (3) strain P-AEG036; (4) strain P-AEGG036. Additional file 5:
**Table S2.** Primers used in this study.
