# Supplementary material for: Modification of targets related to the Entner–Doudoroff/pentose phosphate pathway route for methyl-d-erythritol 4-phosphate-dependent carotenoid biosynthesis in Escherichia coli
Source: Microb Cell Fact. 2015 Aug 12;14:117. doi: 10.1186/s12934-015-0301-x (PMC4534122; doi:10.1186/s12934-015-0301-x)
Supplement: Additional file 1: — Table S1. Biomass and carotenoid production of strains. [file 12934_2015_301_MOESM1_ESM.doc]

Table S1. Biomass and carotenoids production of the strains

|  | Carotenoids Production  (mg/g DCW) | Biomass  (O.D. 600 value) |
| --- | --- | --- |
| Strains in Fig.2 | (neurosporene production) |  |
| W036(*B*) | 2.05±0.1 | 8.7±1.3 |
| P036(*B*) | 2.74±0.1 | 8.7±0.2 |
| KA036 | 2.55±0.1 | 8.2±0.1 |
| KB036 | 2.60±0.1 | 9.6±0.8 |
| KAB036 | 2.66±0.2 | 8.6±0.4 |
| F036 | 1.82±0.1 | 7.6±1.0 |
| Strains in Fig.3 | (neurosporene production) |  |
| W036 | 2.05±0.1 | 8.7±1.3 |
| W-AZ036 | 2.38±0.1 | 9.1±0.4 |
| W-AE036 | 2.75±0.6 | 8.5±0.5 |
| W-AD036 | 1.79±0.1 | 7.6±0.3 |
| Strains in Fig.4 | (neurosporene production) |  |
| W036 | 2.05±0.1 | 8.7±1.3 |
| P036 (*A*)  (*B*) | 2.60±0.2  2.15±0.0 | 9.0±0.2  9.1±0.1 |
| TX036 | 3.23±0.3 | 8.9±0.5 |
| PTX036 | 6.94±0.0 | 14.5±0.0 |
| W-SX036 | 5.03±0.3 | 9.4±0.0 |
| P-SX036 (*A*)  (*B*) | 9.40±0.4  9.09±0.1 | 18.7±0.3  17.8±0.2 |
| TX-SX036 | 5.30±0.5 | 8.4±0.1 |
| PTX-SX036 | 10.71±0.6 | 20.5±0.2 |
| P-AE036 | 5.14±0.0 | 11.6±0.6 |
| P-A-SEX036 | 8.44±0.4 | 18.6±0.1 |
| P-AE-SX036 | 11.44±0.5 | 19.5±0.1 |
| P-AS-SEX036 | 11.14±0.9 | 22.3±0.6 |
| Strains in Fig.6 | (neurosporene production) |  |
| W-SXID036 | 3.81±0.2 | 12.5±1.7 |
| P-SXID036 | 14.66±0.1 | 23.2±0.8 |
| KA-SXID036 | 8.43±1.3 | 17.0±1.0 |
| KAB-SXID036 | 6.88±0.2 | 19.1±0.4 |
| PKAB-SXID036 | 7.21±0.2 | 17.0±0.8 |
| PF-SXID036 | 6.07±0.2 | 19.6±0.3 |
| PY-SXID036 | 11.49±0.2 | 23.2±0.2 |
| Strains in Fig.8 | (lycopene production) |  |
| W-10RK | 1.64±0.1 | 7.5±0.3 |
| TXI-SXID10RK | 4.93±0.1 | 6.6±0.3 |
| PTXI-SXID10RK | 10.43±0.6 | 17.9±0.4 |
| PTXIGK-AEGG-SXID10RK | 17.80±0.6 | 21.4±1.0 |
