# Supplementary figures and images for: Modification of targets related to the Entner–Doudoroff/pentose phosphate pathway route for methyl-d-erythritol 4-phosphate-dependent carotenoid biosynthesis in Escherichia coli
Source: Microb Cell Fact. 2015 Aug 12;14:117. doi: 10.1186/s12934-015-0301-x (PMC4534122; doi:10.1186/s12934-015-0301-x)

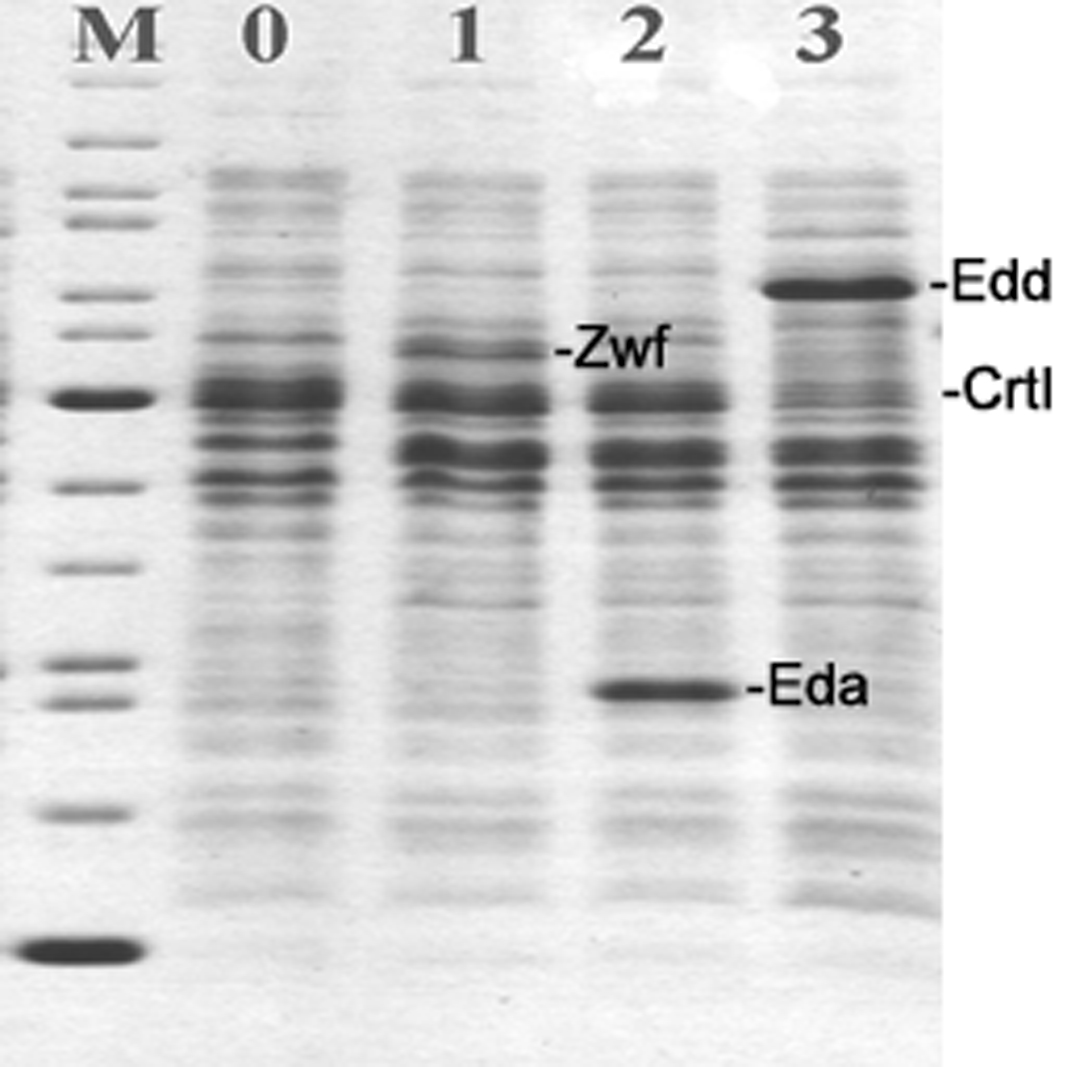

Supplement: Additional file 2: — Figure S1. SDS-PAGE analysis of recombinant proteins produced by Escherichia coli strains: (M) protein markers; (0) strain W036; (1) strain W-AZ036; (2) strain W-AE036; (3) strain W-AD036. [file 12934_2015_301_MOESM2_ESM.jpg]

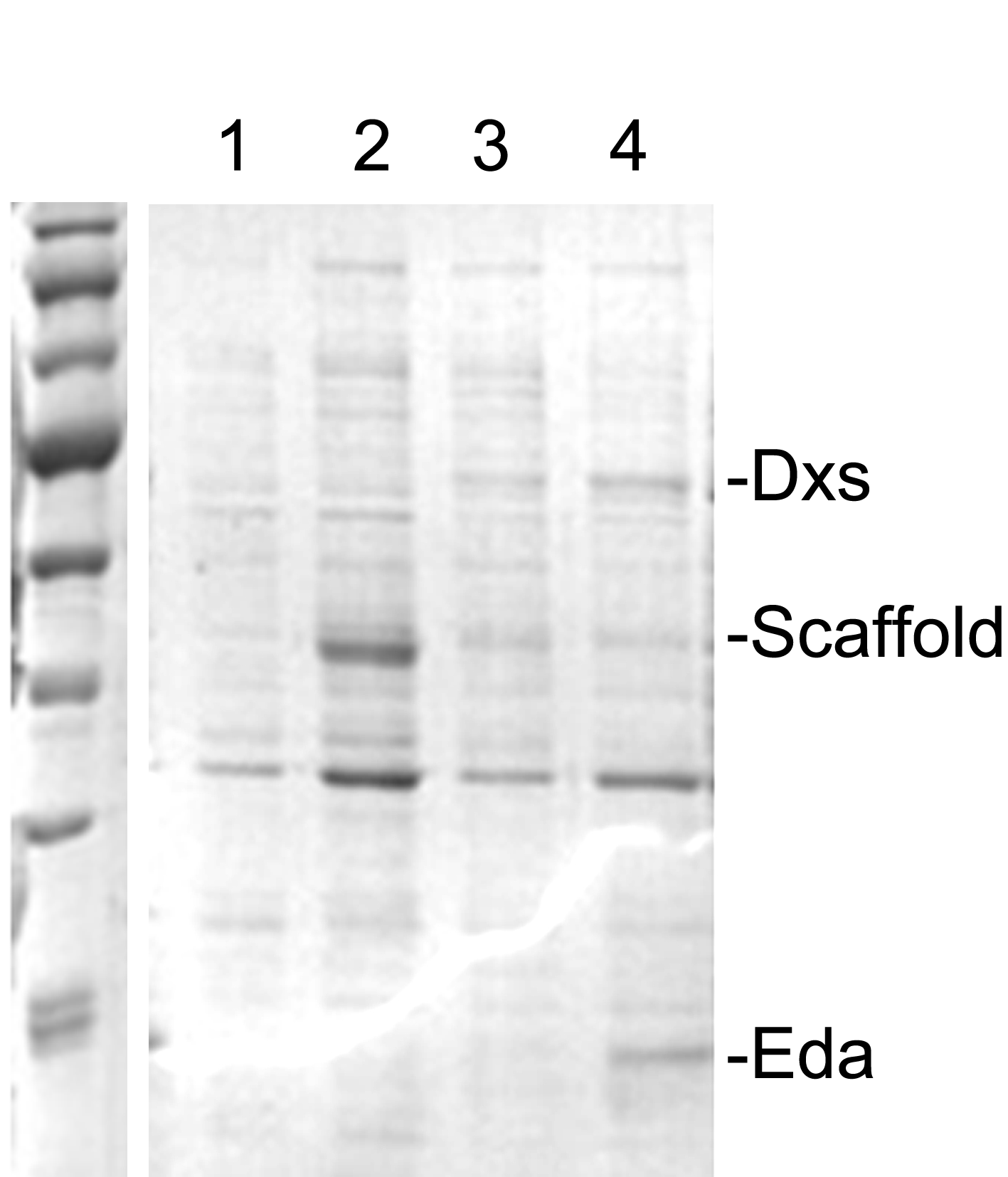

Supplement: Additional file 3: — Figure S2. Results of His-tag pull-down assay. Briefly, strains were induced to express target genes, then cells were lysed by sonication. Cell lysates were centrifuged and supernatants were incubated with Ni–NTA beads for 6 h. Beads were washed to remove unbound proteins. Bound proteins were eluted and analyzed by SDS-PAGE. (M) Markers; (1) strain with pSB1s-eda-dxs (dockerin-fused); (2) strain with pBAD-SS (His-tag fused scaffold protein); (3) strain with pBAD-SS and pSB1s-dxs (dockerin-fused); (4) strain with pBAD-SS and pSB1s-eda-dxs. [file 12934_2015_301_MOESM3_ESM.tif]

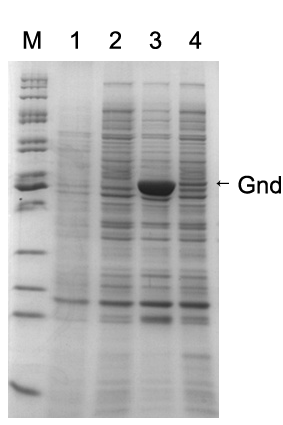

Supplement: Additional file 4: — Figure S3. SDS-PAGE analysis of recombinant proteins produced by Escherichia coli strains: (M) protein markers; (1) strain P036; (2) strain P-AE036; (3) strain P-AEG036; (4) strain P-AEGG036. [file 12934_2015_301_MOESM4_ESM.tif]
