# Supplementary material for: Modification of targets related to the Entner–Doudoroff/pentose phosphate pathway route for methyl-d-erythritol 4-phosphate-dependent carotenoid biosynthesis in Escherichia coli
Source: Microb Cell Fact. 2015 Aug 12;14:117. doi: 10.1186/s12934-015-0301-x (PMC4534122; doi:10.1186/s12934-015-0301-x)
Supplement: Additional file 5: — Table S2. Primers used in this study. [file 12934_2015_301_MOESM5_ESM.doc]

Table S2. Primers used in this study

| Plasmids or strains to be constructed | Primers used | sequence |
| --- | --- | --- |
| pSB1s-*dxs*  pSB1s-*dxs*-*idi-isp*DF | Dxs-F | ACAGTGCCATGGGGAGTTTTGATATTGCCAAATA |
|  | Dxs-R | ACAGTGCTCGAGACCGGCGCGCCACCCCTGCAGGACCGCGGCCGCTTATGCCAGCCAGGCCTTGA |
| pSB1s-*dxs*-*idi-isp*DF | Idi-F | ACAGTGGCGGCCGCCAGGAGGAATTAACCATGCAAACGGAACACGTCAT |
|  | Idi-R | ACAGTGCCTGCAGGTTATTTAAGCTGGGTAAATG |
|  | IspDF-F | ACAGTGCCTGCAGGCAGGAGGAATTAACCATGGCAACCACTCATTTGGA |
|  | IspDF-R | ACAGTGGGCGCGCCTCATTTTGTTGCCTTAATGA |
| pSB1s-*eda*-*dxs*  pBAD-*eda*  pBAD-*eda-gnd*  pBAD-*eda-GCgnd* | Eda-F | ACAGTGCCATGGGGAAAAACTGGAAAACAAGTG |
| pBAD-*eda*  pBAD-*eda-gnd*  pBAD-*eda-GCgnd* | Eda-R | ACAGTGCTCGAGTTACAGCTTAGCGCCTTCTA |
| pBAD-*zwf* | Zwf-F | ACAGTGCCATGGCGGTAACGCAAACAGC |
|  | Zwf-R | CAGTGCTCGAGTTACTCAAACTCATTCCAGG |
| pBAD-*edd* | Edd-F | ACAGTGTCATGAATCCACAATTGTTACG |
|  | Edd-R | ACAGTGCTCGAGTTAAAAAGTGATACAGGTTG |
| pBAD-*eda-gnd* | Gnd-F | CCGCTCGAGAAGGAGATATAATGTCCAAGCAACAGATCGGC |
| pBAD-*eda-gnd*  pBAD-*eda-GCgnd* | Gnd-R | CCGGAATTCTTAATCCAGCCATTCGGTATGG |
| pBAD-*eda-GCgnd* | Gnd-GC-F | CCGCTCGAGAAGGAGATATAATGGGGCCCTCCAAGCAACAGATCGGC |
| pSB1s-*eda*-*dxs* | Eda-Cj-F | CCGCTACCAGTTTAAACCAGCTTAGCGCCT TCTACA |
|  | Eda-Cj-R | TAGAAGGCGCTAAGCTGGTTTAAACTGGTA GCGGTC |
|  | Cj-R | ACAGTGCTCGAGCTAATTGAGCAGTTTAACTT |
|  | Dxs2-F | ACAGTGCTCGAGCAGGAGGAATTAACCATGGGGAGTTTTGATATTGCCAAATA |
|  | Dxs-Ct-F | GAGTACCAGGGCCTGCAGGGCCGGCGAAACCGACGCCGGCCTG |
|  | Dxs-Ct-R | GGCGTCGGTTTCGCCGGCCCTGCAGGCCCTGGTACTCCTTCTAC |
|  | Ct-R | ACAGTGCAGCTGTTAGTTCTTGTACGGCAATGC |
| pBAD-SS | SS-F | CTAGCTAGCATGCTTAAAGTAACAGTAGGAACAGCAG |
|  | SS-R | CTAGTCTAGATTAATGATGATGATGATGATGATCTCCAACATTTACTCCA C |
| pLY036 | An-crtE | ACAGTGCCATGGTAGCAGCTGATAACCT |
|  | An-crtE | ACAGTGGAGCGCTTAGTGATTACGACTAGTGA |
|  | Rs-crtI | ACAGTGGAGCGCAAGGAGATATAATGCCCTCGATCTCGCCCG |
|  | Rs-crtI | ACAGTGAGATCTTCATTCCGCGGCAAGCCTTT |
|  | An-crtB | ACAGTGAGATCTAAGGAGATATAATGCTGCAACTGCCTGATTC |
|  | An-crtB | ACAGTGGAATTCTTATAACACTTGCGATCGCA |
| pLY036/pLY10RK | Idi2-F | ACAGTGGAATTCAAGGAGATATAATGCAAACGGAACACGTCAT |
|  | Idi2-R | ACAGTGCTGCAGTTATTTAAGCTGGGTAAATG |
| pLY10RK | Pag-crtE | ACAGTGCCATGGATATGATGACGGTCTGTGCAGAAC |
|  | Pag-crtE | ACAGTGGAGCGCTTAACTGACGGCAGCGAGTT |
|  | Pan-crtI | ACAGTGGAGCGCAAGGAGATATAATGAAACCAACTACGGTAAT |
|  | Pan-crtI | ACAGTGAGATCTTCATATCAGATCCTCCAGCA |
|  | Pan-crtB | ACAGTGAGATCTAAGGAGATATAATGAATAATCCGTCGTTACT |
|  | Pan-crtB | ACAGTGGAATTCCTAGAGCGGGCGCTGCCAGA |
| pUKM-T5 | T5-F | ACAGTGACTAGTAAGAATCATAAAAAA TTT |
|  | T5-R | ACAGTGGGATCCACCTCCTCTCTCAAATTTA |
| TX | TX-F | GCTTTACAAACGCTGGCGTTCTCGATTTTAAGCGATGCCGATATGCCGGAAGTGTCGGACAGTTCCTATTCCGAAGTTCC |
|  | TX-R | GGTGGAGTCGACCAGTGCCAGGGTCGGGTATTTGGCAATATCAAAACTCATCAGGGGCC ACCTCCTCTCTCAAATTTAT |
| TI | TI-F | TTTTACCTGTCGGCATCCGCTCAAAACGGGCGGTTGTCGATAAACGCTCACTTGGTTAATAGTTCCTATTCCGAAGTTCC |
|  | TI-R | CCCGTGGGAACTCCCTGTGCATTCAATAAAATGACGTGTTCCGTTTGCATAATTTCTCACACCTCCTCTCTCAAATTTAT |
| pS95s-*glk* | Glk-F | ACAGTGCCATGGGGACAAAGTATGCATTAGT |
|  | Glk-R | ACAGTGGAATTCTTACAGAATGTGACCTAAGG |
| pUKM-*glk* | P119 | ACAGTGGGATCCGCATGCCACAGCTAACACCA |
|  | TrrnB | ACAGTGACTAGTAAGGCCCAGTCTTTCGACTG |
| GRK | GRK-F | ACGGGCGTTTTCCGTAACACTGAAAGAATGTAAGCGTTTACCCACTAAGGTATTTTCATGGCATGCCACAGCTAACACCA |
|  | GRK-R | TCGCCAGACCATCGAAGAATTACTGGCGCTGGAATTGCTTTAACTGCGGTTAGTCGCTGGTAGTTCCTATTCCGAAGTTC |
